# Exerkines and cardiometabolic benefits of exercise: from bench to clinic

**DOI:** 10.1038/s44321-024-00027-z

**Published:** 2024-02-06

**Authors:** Leigang Jin, Candela Diaz-Canestro, Yu Wang, Michael Andrew Tse, Aimin Xu

**Affiliations:** 1 State Key Laboratory of Pharmaceutical Biotechnology, The University of Hong Kong, Hong Kong, China; 2 Department of Medicine, The University of Hong Kong, Hong Kong, China; 3 Department of Pharmacology and Pharmacy, The University of Hong Kong, Hong Kong, China; 4 Centre for Sports and Exercise, The University of Hong Kong, Hong Kong, China

**Keywords:** Exercise, Cardiometabolic Disease, Exerkines, Gut Microbiota, Precision Medicine, Cardiovascular System, Digestive System, Metabolism

## Abstract

Regular exercise has both immediate and long-lasting benefits on cardiometabolic health, and has been recommended as a cornerstone of treatment in the management of diabetes and cardiovascular conditions. Exerkines, which are defined as humoral factors responsive to acute or chronic exercise, have emerged as important players conferring some of the multiple cardiometabolic benefits of exercise. Over the past decades, hundreds of exerkines released from skeletal muscle, heart, liver, adipose tissue, brain, and gut have been identified, and several exerkines (such as FGF21, IL-6, and adiponectin) have been exploited therapeutically as exercise mimetics for the treatment of various metabolic and cardiovascular diseases. Recent advances in metagenomics have led to the identification of gut microbiota, a so-called “hidden” metabolic organ, as an additional class of exerkines determining the efficacy of exercise in diabetes prevention, cardiac protection, and exercise performance. Furthermore, multiomics-based studies have shown the feasibility of using baseline exerkine signatures to predict individual responses to exercise with respect to metabolic and cardiorespiratory health. This review aims to explore the molecular pathways whereby exerkine networks mediate the cardiometabolic adaptations to exercise by fine-tuning inter-organ crosstalk, and discuss the roadmaps for translating exerkine-based discovery into the therapeutic application and personalized medicine in the management of the cardiometabolic disease.

## Abstract (web summary)

In this review, A. Xu & colleagues describe the molecular pathways whereby exerkine networks mediate the cardiometabolic adaptations to exercise and discuss the potential translation of exerkine-based discovery into therapeutic application and personalized medicine.

## Graphical abstract

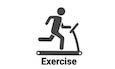

## Introduction

Exercise is one of the most cost-effective strategies for the treatment and prevention of a multitude of chronic disorders, ranging from diabetes, non-alcoholic fatty liver disease (NAFLD), cancer, and cardiovascular dysfunctions, to mental and neurodegenerative diseases. Lifestyle interventions with moderate exercise not only reduce the risk and delay the onset of type 2 diabetes (T2D), but also decrease the incidence of cardiovascular events, disability, and all-cause mortality, with concomitant increases in life expectancy and healthspan (Chakravarty et al, 2008; Knowler et al, 2002; Lieberman et al, 2021). In T2D patients, exercise alone is adequate to improve glycemic control and insulin sensitivity, and lowers cardiovascular risk factors (Hollekim-Strand et al, 2014; Schwingshackl et al, 2014). Understandably, the question of how exercise confers its extensive and profound effects on health has attracted a great deal of research interest throughout the world (Chow et al, 2022).

Six decades ago, it was discovered that blood transferred from working skeletal muscle contained a humoral factor with hypoglycemic properties (Goldstein, 1961). Recent studies have demonstrated that the administration of plasma from exercised mice mediated the cognitive benefits of endurance exercise to older mice and reduced brain inflammation via secreted peptides (De Miguel et al, 2021; Horowitz et al, 2020). It has now become increasingly evident that the primary signaling system responsible for conveying the pleiotropic benefits of exercise is of an endocrine nature (Ruegsegger and Booth, 2018), extending beyond its direct mechanical stimuli (e.g., muscle fiber contraction, elevated central hemodynamics, and vascular wall shear stress). Apart from classical endocrine glands, nearly all the major organs in the body, such as skeletal muscle, adipose tissue, heart, brain, liver, and gut, contribute to the endocrine response to exercise by secreting a bewildering array of bioactive “organokines” into the bloodstream (Lim and Kim, 2023; Minniti et al, 2022). Since the discovery of skeletal muscle-secreted interleukin-6 (IL-6) in 2000 (Steensberg et al, 2000), a growing number of exercise-responsive humoral factors have been identified, including peptide hormones, metabolites, extracellular vehicles, and gut microbes, which are collectively termed as exerkines (Safdar et al, 2016). The exerkine signals can be replicated and manipulated, empowering us to mimic, via pharmacological interventions, the beneficial effects of exercise (Lundby and Robach, 2015).

Since the launch of the Molecular Transducers of Physical Activity Consortium (MoTrPAC) by the National Institutes of Health (NIH), the field of exerkines is rapidly growing to a size that requires specialization, which has been extensively reviewed elsewhere (Robbins and Gerszten, 2023). This review focuses on several exerkines that have been functionally confirmed as crucial molecular transducers, mediating cardiometabolic adaptation to exercise in animals and/or humans. We summarize the molecular pathways whereby these exerkines act in concert to maintain metabolic and cardiovascular homeostasis through interorgan crosstalk, discuss how these exerkines can be therapeutically exploited for the management of cardiometabolic diseases, including the exerkine-based pharmacotherapies and personalized exercise intervention, and highlight the recent clinical trials related to these exerkines.

## Muscle-derived exerkines in cardiometabolic adaptation to exercise

Skeletal muscle, as the largest organ in the human body, plays a vital role in maintaining metabolic homeostasis not only by serving as a primary site for glucose disposal and energy production but also by acting as an endocrine organ that secretes a large number of bioactive myokines with endocrine and/or autocrine functions. Since the discovery of skeletal muscle-secreted IL-6 in response to acute exercise (Febbraio et al, 2004; Pedersen et al, 2003), dozens of myokines have been identified as exerkines (Barros et al, 2022; Chow et al, 2022; Whitham and Febbraio, 2016). However, only a handful of myokines have been functionally characterized in great detail as exerkines mediating the cardiometabolic adaptation to exercise.

**Figure 1:**
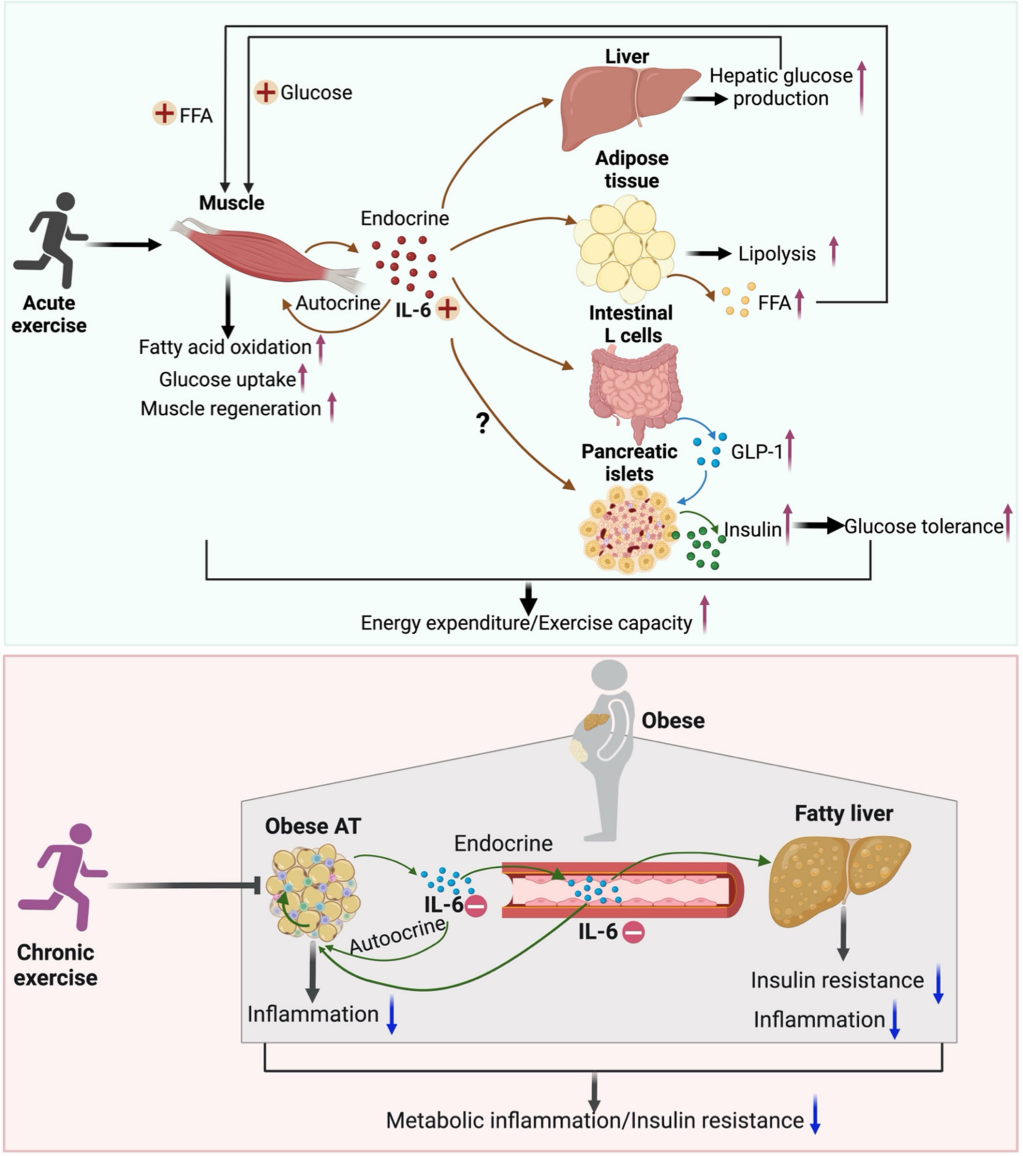
Distinct effects of exercise on skeletal muscle- and adipose-derived IL-6 actions. In response to acute exercise, IL-6 is released from skeletal muscle and acts in an endocrine manner on adipose tissue, liver, gut, and pancreatic islets to mobilize energy supply to skeletal muscle and also in an autocrine manner to facilitate mitochondrial FAO and muscle regeneration, thereby increasing energy expenditure and exercise capacity. In contrast, chronic exercise reduces obesity-associated adipose production of circulating IL-6, thereby attenuating its pro-inflammatory effects in adipose tissues and the liver. AT adipose tissue, FAO fatty acid oxidation, FFA free fatty acid, GLP-1 glucagon-like peptide-1, IL-6 interleukin-6. *Created with BioRender.com*.

***IL-6***, the first identified exercise-responsive myokine, preluded the concept of exerkines (Steensberg et al, 2000). Acute exercise induces a marked elevation of circulating IL-6 from contracting skeletal muscle, which in turn acts in an endocrine or autocrine manner to promote hepatic glucose production in the liver, increase lipolysis in adipose tissue, and enhance fatty acid oxidation and glucose uptake in skeletal muscle (Bertholdt et al, 2017; Carey et al, 2006; Schmidt-Arras and Rose-John, 2016; Wedell-Neergaard et al, 2019). In addition, the elevation of IL-6 levels either by exercise or direct injection, leads to increased production of glucagon-like peptide-1 (GLP-1) by intestinal L cells and pancreatic α-cells, thereby enhancing insulin secretion and improving glucose tolerance (Ellingsgaard et al, 2011). These coordinated effects of IL-6 on multiple organs enable the mobilization of energy supply to contracting skeletal muscle during endurance exercise. Furthermore, IL-6 signaling is required for exercise-induced skeletal muscle regeneration through its actions in muscle-resident regulatory T-cells (Fig. 1) (Becker et al, 2023).

In a randomized, double-blind controlled clinical trial involving people with obesity, blockade of IL-6 signaling through monthly infusions of tocilizumab (an IL-6 receptor antagonist) eliminated the aerobic exercise-induced reduction in epicardial and pericardial adipose tissue, as well as the expected increase in left ventricular mass (Christensen et al, 2019). Similarly, in another randomized placebo-controlled trial involving central obese adults, the reduction in visceral adipose tissue following 12 weeks of endurance exercise training was largely abrogated by IL-6 blockage with tocilizumab (Wedell-Neergaard et al, 2019). On the other hand, improvements in several cardiac functional attributes, such as left ventricular end-diastolic volume, stroke volume, cardiac output, and peak oxygen uptake, remained unaffected by the blockade of IL-6 receptor (Christensen et al, 2019). Therefore, it appears that IL-6 signaling predominantly contributes to the beneficial effects of endurance training on the improvement of obesity and related metabolic outcomes. Based on the findings regarding IL-6 as a beneficial myokine, several genetically engineered IL-6 receptor (gp130) peptide ligands, including human ciliary neurotrophic factor (CNTF) and IC7Fc (a hybrid between IL-6 and CNTF), have been developed. In preclinical studies, both CNTF and IC7Fc potently reduce obesity and related metabolic complications (Findeisen et al, 2019; Gloaguen et al, 1997; Sleeman et al, 2003).

While IL-6 released from skeletal muscle mediates the pleiotropic metabolic benefits of exercise, chronic and excessive production of IL-6 from adipose tissue in obesity contributes to metabolic inflammation and insulin resistance (Han et al, 2020; Makki et al, 2013; Sindhu et al, 2015). Circulating levels of IL-6 are markedly elevated in people with obesity, whereas long-term exercise reduces its circulating levels (Oberbach et al, 2008) (Fig. 1). These apparently paradoxical metabolic effects of IL-6 might be attributed to the intricate and tissue-specific signaling pathways, which involve classical signaling through the interleukin-6 receptor (IL-6R) and gp130 receptor complex, trans-signaling mediated by soluble IL-6R and gp130 complex, and cluster signaling via binding to IL-6R and gp130 from two different types of cells (Baran et al, 2018; Jones et al, 2011; Tanaka et al, 2014).

Therefore, the overall metabolic effects of this cytokine are influenced by the magnitude and duration of IL-6 elevation, as well as soluble IL-6R levels, thereby leading to its signaling activation in distinct sets of cells/organs under different pathophysiological conditions. In support of this notion, data obtained from mice with cell-type-specific knockout or knockdown of *IL-6R* suggest divergent metabolic roles of IL-6 signaling in different types of cells. For example, mice with hepatocyte-specific *IL-6RA* deficiency displayed systemic insulin resistance and notable hepatic inflammation even under a normal diet (Wunderlich et al, 2010). Similarly, myeloid cell-specific *IL-6R*-deficient obese mice exhibited a more adverse metabolic profile compared to obese wild-type littermates, associated with an increased shift of macrophages to a pro-inflammatory M1 phenotype (Braune et al, 2017; Mauer et al, 2014). Conversely, T cell-specific or natural killer cell-specific *IL-6R* deficiency ameliorated insulin resistance in obese mice (Theurich et al, 2017; Xu et al, 2017), indicating that IL-6 can exert both pro-inflammatory and anti-inflammatory actions.

***Myonectin***, also named as C1q TNFα-related protein isoform 15 (C1QTNF15), shares a similar domain organization with adiponectin and was first identified in myotubes as a myokine (Ouchi and Walsh, 2012; Seldin et al, 2012). A subsequent study found that 1-week of aerobic exercise increases plasma levels of myonectin in both healthy and obese women (Lim et al, 2012; Pourranjbar et al, 2018). In mice, skeletal muscle-derived myonectin is induced by exercise, which in turn contributes to cardiac and metabolic adaptations. Endurance training reduces infarct size after ischemia/reperfusion (I/R) in wild-type but not in *myonectin*-knockout mice, whereas transgenic overexpression of *myonectin* alone is sufficient to attenuate myocardial damage after I/R (Otaka et al, 2018). Furthermore, myonectin protects against skeletal muscle dysfunction and atrophy through the activation of AMP-activated protein kinase/proliferator-activated receptor co-activator-1α (AMPK/PGC1α) signaling pathway in mice (Ozaki et al, 2023). Skeletal muscle-derived myonectin has been functionally linked to systemic lipid homeostasis through its actions in the liver and adipose tissues (Seldin et al, 2012). However, whether myonectin mediates the metabolic adaptation to exercise has yet to be determined. Further studies in humans are needed to assess the effects of exercise on myonectin and its potential mediation of cardiac and metabolic adaptations to exercise.

***Musclin***, a myokine with high homology to natriuretic peptides (NP), has emerged as an important mediator for mitochondrial adaptations to endurance training (Subbotina et al, 2015). In mice, both the mRNA expression and circulating level of musclin are obviously elevated in response to treadmill exercise, but genetic ablation of the *musclin*-encoding gene compromises aerobic exercise capacity due to reductions in exercise-induced mitochondrial protein contents, respiratory complex protein expression, and succinate dehydrogenase activity in skeletal muscles (Nishizawa et al, 2004; Subbotina et al, 2015). In contrast, replenishment with recombinant musclin restores mitochondrial oxidative capacity and aerobic exercise capacity in endurance-trained *musclin*-knockout mice compared to wild-type mice. *Musclin*-deficient mice also exhibit impairment in exercise-induced cardiac protection against ischemic injury, whereas infusion of a synthetic musclin peptide to a level comparable to that observed after exercise mimics the cardioprotective effect of exercise in sedentary mice (Harris et al, 2023). Likewise, pressure overload-induced cardiac dysfunction is exacerbated by skeletal muscle-specific disruption of the *musclin* gene, but it is mitigated by its overexpression in skeletal muscle (Szaroszyk et al, 2022). Mechanistically, musclin induces the expression of C-type natriuretic peptide (CNP) possibly by competitive binding to the NP clearance receptor (NPR-C), thereby promoting cardiomyocyte contractility through protein kinase A and inhibiting fibroblast activation through protein kinase G (PKG) signaling (Kita et al, 2009; Szaroszyk et al, 2022). Activation of PKG also contributes to the effect of musclin in mediating exercise-induced mitochondrial biogenesis through cAMP-dependent induction of the transcription co-activator PGC1α (Subbotina et al, 2015). In patients with heart failure, both the serum level and skeletal muscle expression of musclin are decreased compared with healthy controls (Szaroszyk et al, 2022). Taken together, these experimental and clinical data support the role of skeletal muscle-released musclin as a critical mediator of exercise-induced remodeling in cardiac and skeletal muscle. There is, however, scarce clinical data available regarding changes in circulating musclin levels in response to exercise in humans. Although musclin has been shown to attenuate lipid deposition in hepatocytes (Cho et al, 2023), it paradoxically inhibits biogenesis and thermogenesis of beige adipocytes in mice (Jin et al, 2023a), Hence, it remains unclear whether musclin participates in the metabolic adaptation to exercise.

There are several other emerging exercise-responsive myokines worthy of mention, including apelin, which mediates exercise-induced reversal of age-associated sarcopenia by promoting mitochondrial biogenesis in skeletal muscle (Vinel et al, 2018), and β-aminoisobutyric acid (BAIBA) and irisin, both of which have been implicated in the browning of white adipose tissue (WAT) (Roberts et al, 2014; Zhang et al, 2018). However, the effect of exercise on browning and thermogenesis of WAT has been controversial, and the roles of these exerkines in cardiometabolic adaptations to exercise require further exploration (Chow et al, 2022).

## Liver- and adipose-derived exerkines in cardiometabolic protection

The liver and adipose tissues secrete a large number of hepatokines and adipokines critical for the regulation of glucose/lipid metabolism and cardiovascular homeostasis (Meex and Watt, 2017; Scheja and Heeren, 2019; Stefan et al, 2023; Yoo and Choi, 2015). Aberrant production and/or function of hepatokines and adipokines are important contributors to obesity-related cardiometabolic diseases (Meex and Watt, 2017; Robbins and Gerszten, 2023; Scheja and Heeren, 2019; Stefan et al, 2023; Yoo and Choi, 2015). Emerging data from both preclinical and clinical studies has identified a significant portion of hepatokines and adipokines responsive to exercise intervention. However, only a few of them have been functionally linked to the cardiometabolic effects of exercise, including adiponectin (Caldwell et al, 2021; Lee et al, 2011), fibroblast growth factor 21 **(**FGF21) (Bo et al, 2023; Jin et al, 2022; Ma et al, 2021), 12,13-dihydroxy-9Z-octadecenoic acid (12,13-diHOME) (Stanford et al, 2018), and carboxylesterase 2 (CES2) (Wei et al, 2023).

***Adiponectin***, an adipokine predominantly secreted from adipocytes, exerts its multiple salutary effects (insulin-sensitization, anti-inflammation, cardiometabolic and neuronal protection) by binding to the receptors adipoR1, adipoR2, or T-cadherin (Kadowaki et al, 2006; Maeda et al, 2002; Scheja and Heeren, 2019). These adiponectin receptors are widely expressed in almost all tissues, conferring the pleiotropic benefits of adiponectin through activation of multiple intracellular signaling pathways such as AMPK and peroxisome proliferator-activated receptor-α (PPARα) (Yamauchi and Kadowaki, 2008). Unlike most adipokines, plasma levels of adiponectin are markedly decreased in obesity and its related metabolic complications, and are inversely associated with glucolipid profiles and insulin sensitivity (Achari and Jain, 2017; Hui et al, 2012). Longitudinal studies across different ethnic groups have consistently identified low adiponectin as an independent predictor for T2D and NAFLD (Savvidou et al, 2009; Spranger et al, 2003). In animals, *adiponectin* deficiency exacerbates obesity-related insulin resistance and cardiometabolic syndrome, whereas replenishment with adiponectin or its receptor agonists (such as AdipoRon) has been shown to reverse these conditions (Kadowaki and Yamauchi, 2005; Xu et al, 2003; Yamauchi et al, 2001). Elevated circulating adiponectin levels have been observed in both healthy individuals and people with obesity as well as rodent models in response to different types of exercise interventions (Bouassida et al, 2010; Geng et al, 2019; Kriketos et al, 2004; Yang et al, 2019). A meta-analysis, including data from eight trials, demonstrated that aerobic exercise significantly increased serum adiponectin concentrations in children and adolescents with obesity in an intensity-dependent manner (Zhang et al, 2023). In mice, the role of adiponectin as a critical mediator of exercise in improving cardiovascular function is supported by the finding that treadmill exercise-induced increases in ejection fraction, fractional shortening, and coronary flow or acetylcholine-mediated endothelium-dependent vasodilation are largely abrogated by genetic ablation of *adiponectin* (Caldwell et al, 2021; Lee et al, 2011). Furthermore, *adiponectin* deficiency compromises acute exercise-induced hippocampal neurogenesis and anti-depressant effects in both stressed and diabetic mice, and also impairs the ability of exercise to activate AMPK and IL-6 secretion in skeletal muscle (Diniz et al, 2019; Yau et al, 2014). In contrast, the adiponectin agonists mimic the effects of exercise on protection against several obesity-related complications, including alleviation of glucose intolerance and insulin resistance (Nicolas et al, 2020), reduction of liver steatosis and inflammation in mouse models of NAFLD (Xu et al, 2020), and preservation of cognitive functions and neural plasticity in diabetic mouse model (Lee et al, 2021).

***FGF21***, a peptide hormone mainly secreted by the liver, is currently garnering increasing attention due to its potential role in mediating cardiometabolic adaptations to exercise training. Preclinical studies have consistently demonstrated the multiple potent therapeutic benefits of FGF21 for a cluster of obesity-related cardiometabolic disorders, including the reduction in adiposity and improvement in insulin resistance, hyperglycemia, dyslipidemia, NAFLD, atherosclerosis, and heart dysfunction (Geng et al, 2020; Jin et al, 2023b). FGF21 exerts its actions by binding to its receptor complex, comprising fibroblast growth factor receptor-1 (FGFR1) and the co-receptor β-Klotho (KLB), the latter of which is expressed only in the brain, adipose tissue, liver, and pancreas, and therefore determines the target selectivity of FGF21 (Ge et al, 2011; Goetz et al, 2007). In mice, FGF21 induces adipocyte production of adiponectin, which in turn acts as an indispensable downstream effector, conferring protective effects of FGF21 against obesity-induced metabolic complications and atherosclerosis (Holland et al, 2013; Lin et al, 2015; Lin et al, 2013). Likewise, several recent phase-2b clinical trials have shown the remarkable effects of long-acting FGF21 analogs in decreasing liver fat content, inflammation, fibrosis, and dyslipidemia in individuals with obesity who have biopsy-confirmed NASH, accompanied by obvious elevations in plasma adiponectin levels (Abdelmalek et al, 2021; Bhatt et al, 2023; Loomba et al, 2023).

**Figure 2:**
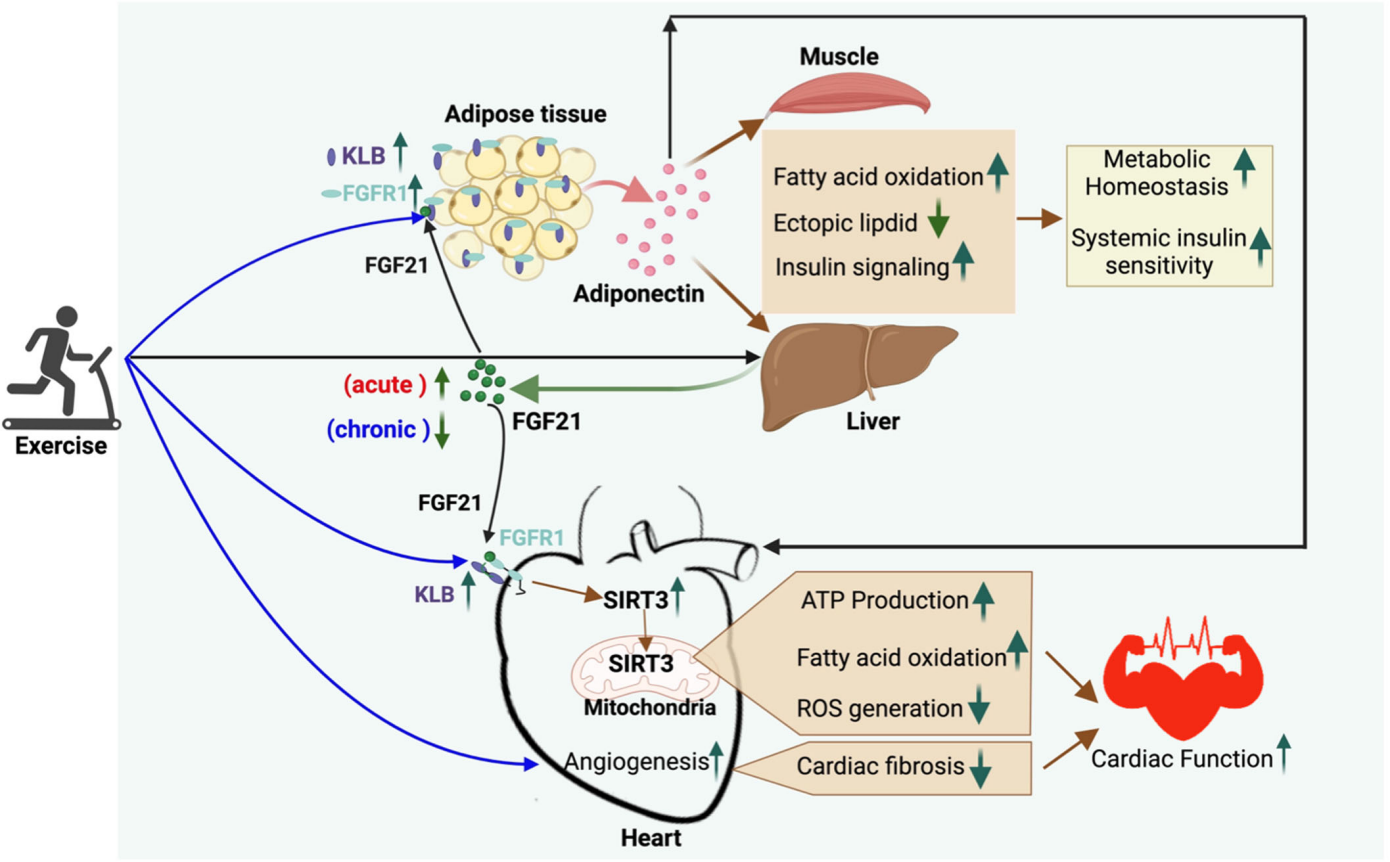
The FGF21-adiponectin axis confers the effects of exercise on cardiometabolic protection via interorgan crosstalk. Acute exercise increases circulating FGF21, whilst chronic exercise induces the expression of the FGF21 co-receptor KLB in the adipose tissue and heart, leading to increased FGF21 sensitivity. In adipose tissue, enhanced FGF21 actions lead to augmented secretion of adiponectin, which exerts its endocrine actions on skeletal muscle and liver enhancing fatty acid oxidation, mitigating ectopic lipid accumulation, and thereby increasing insulin sensitivity and metabolic homeostasis. In the exercised heart, FGF21 signaling induces the expression of mitochondrial deacetylase SIRT3, which in turn improves cardiac functions by promoting fatty acid oxidation, ATP production, and reducing ROS. Furthermore, FGF21 mediates the protective effects of exercise against myocardial infarction and cardiac fibrosis. FGF21-induced secretion of adiponectin may also contribute to cardiac protection by exercise. ATP adenosine triphosphate, FGF21 fibroblast growth factor 21, FGFR1 fibroblast growth factor receptor-1, KLB β-Klotho, ROS reactive oxygen species, SIRT3 sirtuin-3. *Created with BioRender.com*.

In both humans and rodents, circulating levels of FGF21 are increased after acute exercise, but decreased after long-term exercise (>4 weeks) due to increased FGF21 sensitivity (Fletcher et al, 2012; Kim et al, 2013; Porflitt-Rodriguez et al, 2022; Yang et al, 2011). It has been proposed that elevated serum FGF21 levels in individuals with obesity and rodents result from FGF21 resistance in adipose tissues due to reduced expression of its receptor complex (Fisher et al, 2010; Geng et al, 2019; Li et al, 2018). FGF21-evoked extracellular signal-related kinases 1/2 (ERK1/2) phosphorylation and adiponectin secretion in adipocytes, as well as the effects of FGF21 on lowering glucose and insulin, are impaired in dietary obese mice, while chronic exercise restores these FGF21 actions by inducing adipose FGFR1 and KLB expression via PPARγ (Geng et al, 2019; Yang et al, 2019). Furthermore, the beneficial effects of exercise on the alleviation of obesity-associated insulin resistance, glucose intolerance, and ectopic lipid accumulation are abrogated in adipocyte-specific *Klb* knockout and *Fgf21* deficient mice (Geng et al, 2019; Loyd et al, 2016), supporting an obligatory role of FGF21 signaling in adipose tissue for the metabolic benefits of exercise (Fig. 2). Similarly, although cardiomyocytes are not the direct target of FGF21 due to the lack of KLB expression in sedentary mice, cardiac KLB expression is drastically induced by endurance exercise (Jin et al, 2022), thus making the heart an FGF21 responsive organ. In turn, the increased cardiac FGF21 sensitivity induced by endurance exercise prevents diabetic cardiomyopathy by activating the (nicotinamide adenine dinucleotide) NAD^+^-dependent deacetylase sirtuin-3 (SIRT3) and preserving mitochondrial integrity via the AMPK-(forkhead box protein O3) FOXO3 signaling pathway (Jin et al, 2022). Both cardiomyocyte-selective *Klb* and *Fgf21* knockout mice are resistant to exercise in cardioprotection. Likewise, *Fgf21* deficiency in mice compromises the effects of aerobic exercise on promoting myocardial angiogenesis and alleviating cardiac fibrosis induced by ischemic injury (Bo et al, 2023; Ma et al, 2021). Therefore, these findings consistently support the notion that the selective increase in FGF21 sensitivity in adipose and cardiac tissues is an important mechanism mediating the cardiometabolic benefits of FGF21 (Fig. 2), and also suggest that FGF21-based pharmacotherapy for obesity-related cardiometabolic diseases should be implemented in conjunction with exercise intervention to increase its sensitivity.

Apart from the FGF21-adiponectin axis, emerging evidence suggests brown adipose tissue-derived 12,13-diHOME, and liver-secreted CES2 as exercise-responsive adipokines/hepatokines conferring the cardiometabolic effects of exercise (Pinckard et al, 2021; Wei et al, 2023). Circulating 12,13-diHOME level is decreased in patients with cardiovascular disease, whereas acute exercise increases 12,13-diHOME secretion from brown adipose tissue (BAT) into the bloodstream (Pinckard et al, 2021; Vasan et al, 2019). Elevated 12,13-diHOME, in turn, acts on skeletal muscle and heart to increase oxidative capacity and to enhance mitochondrial respiration and cardiac hemodynamics, respectively, through nitric oxide synthase type 1 (Pinckard et al, 2021; Stanford et al, 2018). In a recent study using a cell-type-specific secretome mapping approach, Wei et al discovered soluble CES2 as a novel exercise-responsive hepatokine with anti-obesity and anti-diabetic actions as well as endurance-enhancing effects (Wei et al, 2023). This is consistent with previous studies demonstrating that hepatocyte-specific overexpression of *CES2* improved glucose tolerance and insulin sensitivity and attenuated NAFLD (Li et al, 2016; Xu et al, 2021). However, further studies are needed to explore the physiological roles and clinical relevance of 12,13-diHOME and CSE2 in cardiometabolic adaptation to exercise.

While the aforementioned studies support the obligatory role of FGF21 signaling in mediating the cardiometabolic benefits of exercise, another study showed that *Fgf21* deficiency in mice resulted in compromised effects of voluntary exercise in induction of *PGC1*α gene expression, citrate synthase activity, cytochrome c oxidase I (COX I), and total AMPK content, but did not affect the anti-inflammatory effects of exercise in WAT (Porter et al, 2017). However, it is important to note that the standard chow-fed lean mice, which did not exhibit obvious obesity-related metabolic inflammation and dysfunction, were used in this study, and the wild-type controls were not from the littermates of *Fgf21* KO mice.

## Gut microbiota as emerging molecular transducers of exercise

Both cross-sectional studies and randomized controlled trials have shown that exercise induces compositional and functional changes in gut microbiota, a “hidden” metabolic organ comprising approximately 40 trillion microbial cells (Aya et al, 2023; Chen et al, 2023; Dziewiecka et al, 2022; Wang et al, 2023). Professional athletes exhibit much higher gut microbial diversity than sedentary subjects, characterized by increased pathways related to amino acids and carbohydrate metabolism (Barton et al, 2018; Clarke et al, 2014; O’Brien et al, 2022). Aerobic exercise induces compositional and functional changes in the gut microbiota of both lean and obese subjects independent of diet (Allen et al, 2018), which are reversed with training cessation. However, due to the complexity of gut microbiota and its vulnerability to environmental, dietary, and geographical factors, the data published in different studies are highly variable, and only a few studies have addressed the role of specific microbial species in cardiometabolic adaptation to exercise.

Scheiman et al identified a lactate-metabolizing microbiome, i.e., *Veillonella atypica*, that increases after exercise in two independent cohorts of marathoners and Olympic trial rowers (Scheiman et al, 2019). Inoculation of *Veillonella atypica* into mice significantly increases exhaustive treadmill run time by metabolizing lactate into the short-chain fatty acids (SCFAs) acetate and propionate (Scheiman et al, 2019), which, in turn, augments energy supply and enhances mitochondrial oxidative capacity (Marttinen et al, 2020). Morita and colleagues found that the increased abundance of fecal *Bacteroides Uniformis* (*B. Uniformis)* is negatively associated with 3000 m of race time in Japanese long-distance runners (Morita et al, 2023). *B. Uniformis* administration in mice increases swimming time to exhaustion, accompanied by elevated SCFAs concentrations and expression of gluconeogenic genes but decreased glycogen contents in the liver. Supplementation of α-cyclodextrin, a preferred substrate of *B. Uniformis*, increases bacterial abundance and endurance exercise performance in healthy men (Morita et al, 2023). Furthermore, exercise enhances the gut microbial production of fatty acid amides, which in turn stimulates sensory neuron activity and elevates dopamine levels in the ventral striatum during exercise, thereby improving exercise motivation and performance (Dohnalova et al, 2022).

**Figure 3:**
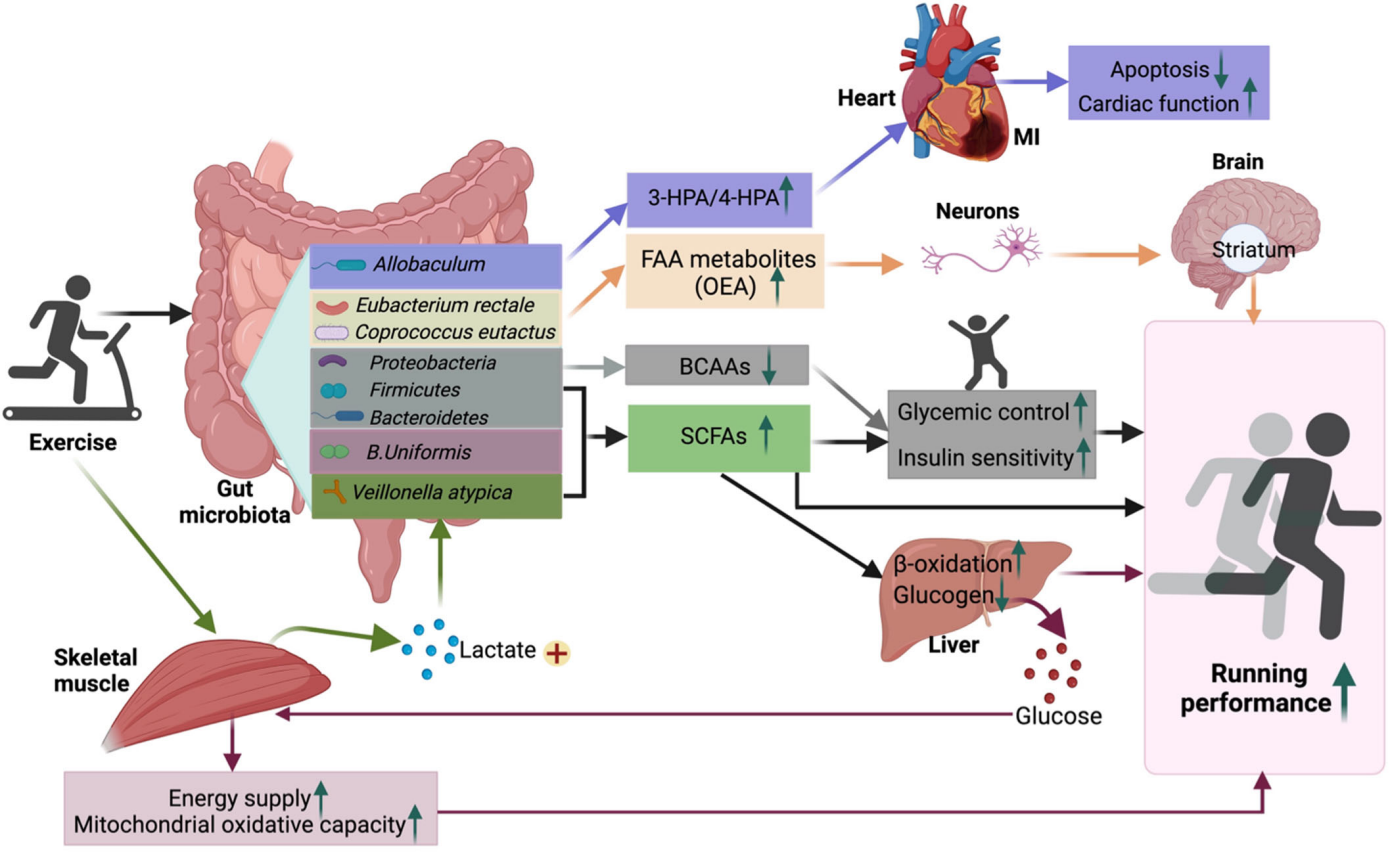
Gut microbiota mediates the effects of exercise on cardiometabolic health and physical performance. Exercise induces structural and functional changes in gut microbiota (such as *Proteobacteria*, *Firmicutes*, *Bacteroidetes*, and *B. Uniformis*), shifting the microbial fermentation of carbohydrates towards decreased production of BCAAs but increased SCFAs, which in turn improves insulin sensitivity and glycemic homeostasis. Furthermore, the enriched abundance of *Veillonella atypica* also contributes to elevated production of SCFAs by metabolizing skeletal muscle-derived lactate, thereby leading to increased energy supply and enhanced exercise capacity. Additionally, exercise enhances the production of fatty acid amides (e.g., OEA) by specific gut microbiota (*Eubacterium rectale* and *Coprococcus eutactus*). OEA, in turn, stimulates sensory neuron activity leading to improved motivation and performance of exercise. Furthermore, exercise increases the production of 3-HPA and 4-HPA by particular microbes (e.g., *Allobaculum*), thus protecting against myocardial infarction-induced heart failure. 3-HPA 3-hydroxyphenylacetic acid, 4-HPA 4-hydroxybenzoic acid, BCAAs branched-chain amino acids, FFA free fatty acids, MI myocardial infarction, OEA oleoylethanolamide, SCFAs short-chain fatty acids. *Created with BioRender.com*.

In a randomized, placebo-controlled clinical trial that included 39 medication-naive men with overweight and prediabetes, improvement in glucose metabolism and insulin sensitivity in response to a 12-week high-intensity interval training (HIIT) was closely associated with changes in six microbial species belonging to *Firmicutes, Bacteroidetes*, and *Proteobacteria* (Liu et al, 2020). Notably, there was an obvious segregation in exercise-reshaped metabolic activities of gut microbiota between exercise responders and non-responders with respect to insulin sensitivity: the microbiome of responders exhibited an enhanced capacity for SCFAs biosynthesis and catabolism of branched-chain amino acids, whereas those of non-responders were characterized by increased production of detrimental metabolites. Fecal microbial transplantation from exercise responders, but not non-responders, mimicked the effects of exercise on alleviation of insulin resistance and glucose intolerance in obese mice, suggesting that gut microbiota itself is sufficient to confer the metabolic benefit of exercise in diabetes prevention (Liu et al, 2020). Intriguingly, replenishment with SCFAs in mice colonized with microbiota from non-responders rescued the non-responsiveness in glucose homeostasis and insulin sensitivity, raising the possibility of using dietary intervention to restore exercise efficacy in those non-responders. Similarly, transplantation of fecal microbiota from exercised mice has been shown to improve cardiac function after myocardial infarction as compared to those mice receiving microbiota transplants from non-exercised mice, possibly by modulating the production of 3-hydroxyphenylacetic acid (3-HPA) and 4-hydroxybenzoic acid (4-HPA) (Zhou et al, 2022). Furthermore, baseline gut microbial signatures have been shown to be strong predictors of individual responses to exercise with regards to the reduction of liver fat and insulin resistance in two independent, randomized controlled trials (Cheng et al, 2022; Liu et al, 2020). Therefore, gut microbiota not only influences exercise performance but also determines individual responses and imparts the cardiometabolic effects of exercise (Fig. 3). Nevertheless, it remains largely unknown how the exercise-shaped microbiome interacts with the host to confer its health benefits. In this regard, a recent Olink-based proteomic profiling in men with overweight has observed a close association between changes in plasma proteins involved in gastro-intestinal mucosal immunity (such as gut-secreted trefoil factor-2) and metabolic outcomes in response to HIIT (Diaz-Canestro et al, 2023). These findings raise the possibility that gut microbiota may confer the systemic effects of exercise by modulating gut-derived hormones, albeit further investigation is still warranted.

Emerging evidence suggests that individual responsiveness to exercise intervention, in terms of improving insulin sensitivity and glycaemic control, can be predicted by a machine-learning algorithm integrating baseline signatures of gut microbiome and metabolites (Liu et al, 2020) or circulating exerkine profiles (Diaz-Canestro et al, 2023). These algorithms can be potentially exploited for the implementation of personalized exercise intervention. For those non-responders, additional dietary/nutritional interventions, such as supplementation with probiotics/prebiotics and SCFAs, are recommended to restore exercise efficiency in the prevention of cardiometabolic diseases.

## Concluding remarks and future perspectives

Research efforts on peptide exerkines and their signaling pathways have contributed to the development of several therapeutic leads for cardiometabolic diseases, including the IL-6-like molecule IC7Fc, adiponectin mimetics (such as AdipoRon), and long-acting FGF21 analogs, the latter of which are under phase-2 clinical trials (Abdelmalek et al, 2021; Bhatt et al, 2023; Loomba et al, 2023). The discovery of gut microbiota and microbial metabolites as the “hidden” molecular transducers of exercise paves the new way to develop edible exercise mimetics (such as prebiotics and/or probiotics) for improving exercise performance and cardiometabolic health by targeting exercise-responsive microbes and microbial metabolites, as evidenced in several clinical studies (Cheng et al, 2022; Wang et al, 2023). Furthermore, baseline exerkine signatures have been shown to predict individual responses to exercise, potentially guiding the implementation of personalized exercise interventions for cardiometabolic diseases. The knowledge from exerkine research also provides solid scientific evidence for the synergism between lifestyle intervention and pharmacotherapy in the management of cardiometabolic diseases, as exemplified by the finding that chronic exercise selectively increases FGF21 sensitivity in its target tissues, thus making FGF21-based pharmacotherapy more effective.

Recent advances in multiomics technologies have enabled comprehensive, unbiased profiling of molecular choreography in response to both acute and chronic exercise, revealing hundreds of previously unappreciated exerkines closely associated with cardiorespiratory fitness, insulin sensitivity, metabolic outcomes, or exercise performance (Contrepois et al, 2020; Diaz-Canestro et al, 2023; Mi et al, 2023; Robbins and Gerszten, 2023). While these findings provide exciting opportunities for expanding exerkine research and drug discovery, they also bring many challenges. Firstly, the longitudinal trajectory of exerkine changes varies greatly across different studies, influenced by the mode, intensity, duration of exercise, and other unknown factors. An international consortium with unified approaches is needed to construct reproducible, standardized, holistic maps for dynamic changes of the exerkine network linked to the phenomics dataset. Secondly, the complex exerkine regulatory network and the reciprocal interactions among exerkines in coordinating interorgan crosstalk are another important research area for further exploration. Thirdly, there is also large interpersonal variability in exerkine changes in response to the same type of exercise, with many exerkines exhibiting opposite directions in exercise responders and non-responders. Further investigation into these variable exerkines may help to understand the phenomenon of “exercise resistance” (Bell et al, 2022; Diaz-Canestro et al, 2023), paving the way for personalized exercise intervention.

Another important missing research gap in this field pertains to the sex difference in exerkine production. Furthermore, there is a lack of comparison regarding the effects of different exercise modalities and intensities on exerkine production. In this regard, the MoTrPAC project (Sanford et al, 2020), which includes both male and female subjects participating in both resistance and endurance exercise programs, is expected to provide valuable information on the effects of gender, exercise modality, and intensity on exerkine production. Finally, among hundreds of exerkines identified so far, only a few exerkines have been mechanistically linked to the health benefits of exercise. The physiological function of the majority of exerkines remains largely unexplored in the context of exercise. An integrated approach that combines the traditional reductionist strategy with functional genomics and phenomics should be exploited to dissect the complex, dynamic exerkine regulatory network functionally involved in the cardiometabolic adaptation to exercise.

## Pending issues

1. Elucidate the physiological roles and functional mechanisms for a large number of newly identified exerkines.
2. Assess how gender, age, exercise modality, intensity, and duration differentially modulate exerkine production.
3. Dissect the complex exerkine regulatory network and their roles in mediating interorgan crosstalk under various pathophysiological conditions.
4. Construct a comprehensive multiomics map linking changes in exerkines to various clinical outcomes in humans.
5. Optimize and implement exerkine-based strategies for predicting and monitoring the individual responsiveness to different types of exercise.
6. Develop exercise mimetics as potential pharmacotherapies for cardiometabolic diseases by targeting exerkine pathways.

## Glossary

Exercise non-responders: Individuals who do not experience on significant improvement following an exercise intervention training. For example, a large proportion of individuals, ranging from 7 to 69%, do not respond (non-responders) or even respond adversely to exercise in terms of insulin sensitivity and glucose homeostasis. Genetic, epigenetic factors, and gut microbiome have been implicated as potential contributing factors to exercise non-responsiveness.
Exerkines: Also known as exercise-responsive factors, are defined as signaling molecules released from a multitude of organs in response to acute and/or chronic exercise. These moieties encompass a wide range of bioactive substances, including peptide hormones, cytokines, metabolites, nucleic acids, extracellular vesicles, and gut microbes, and exert their effects through endocrine, paracrine, and autocrine pathways.
FGF21 resistance: A pathological condition characterized by elevated circulating level of the peptide hormone FGF21 and blunted responses of cells to FGF21. This phenomenon has been observed in animal models with obesity and is attributed to reduced expression of the FGF21 receptor complex comprising of FGFR1 and β-klotho.
Olink proteomic profiling: A quantitative proteomics method based on proximity extension assay (PEA) technology, which uses DNA oligonucleotide-labeled antibody pairs to bind target proteins. The oligonucleotide pairs hybridize and are extended by DNA polymerase to create a unique DNA barcode, subsequently read out using PCR or next-generation sequencing. It can measure 92 to ~5000 proteins simultaneously from a few microliters of samples with high specificity and sensitivity.
Organokines: A group of bioactive molecules secreted from different organs that play vital roles in coordinating interorgan crosstalk to maintain body homeostasis. It includes myokines, cardiokines, hepatokines, adipokines, and neurokines secreted from skeletal muscle, heart, liver, adipose tissues, and nervous system, respectively.
